# Spray-Dried Multiple Emulsions as Co-Delivery Systems for Chlorogenic Acid and Curcumin

**DOI:** 10.3390/antiox14101257

**Published:** 2025-10-20

**Authors:** Javier Paredes-Toledo, Javier Herrera, Estefanía González, Paz Robert, Begoña Giménez

**Affiliations:** 1 Department of Food Science and Technology, Faculty of Technology, University of Santiago of Chile, Av. Víctor Jara 3769, Estación Central 9170124, Santiago, Chile; javier.paredes@usach.cl (J.P.-T.); javier.herrera.c@usach.cl (J.H.); 2 School of Health, Universidad de O’Higgins, Av. Libertador Bernardo O’Higgins 611, Rancagua 2820000, Cachapoal, Chile; estafania.gonzalez@uoh.cl; 3 Department of Food Science and Chemical Technology, Faculty of Chemical and Pharmaceutical Sciences, University of Chile, Santos Dumont 964, Independencia 8380494, Santiago, Chile; proberts@uchile.cl

**Keywords:** chlorogenic acid, curcumin, multiple emulsions, spray drying, in vitro gastrointestinal digestion

## Abstract

The low stability and bioaccessibility of polyphenols limit their application in functional foods. To address this, chlorogenic acid (CGA) and curcumin (CU) were selected as model compounds and co-encapsulated in spray-dried linseed oil (LO) multiple emulsions (MEs), using octenyl succinic anhydride-modified waxy maize starch as encapsulating agent. Water-in-oil-in-water MEs were prepared by two-step high-pressure homogenization and spray-dried under optimized conditions determined by response surface methodology to minimize surface oil. The resulting microparticles were characterized for encapsulation efficiency (EE), morphology, oxidative stability, and performance under simulated gastrointestinal digestion (INFOGEST protocol). Both CGA and CU exhibited high EE in microparticles (~88–90%), with spray drying significantly improving CGA retention compared to liquid emulsions. Microparticles also showed improved oxidative stability due to the presence of antioxidants. During digestion, CU bioaccessibility decreased (62.7%) relative to liquid MEs (83.6%), consistent with reduced lipid digestion. Conversely, CGA bioaccessibility was higher in microparticles (47.6%) than in MEs (29.2%), indicating a protective effect of the encapsulating agent under intestinal conditions. Overall, spray drying stabilized linseed oil-based MEs and enabled effective co-encapsulation of hydrophilic and lipophilic compounds, supporting their potential as multifunctional delivery systems for functional foods.

## 1. Introduction

Polyphenols are secondary plant metabolites that have been widely investigated in recent decades due to their broad biological functions and their potential to lower the risk of chronic disorders, including cancer, cardiovascular diseases, diabetes, and neurodegenerative conditions [1,2]. These recognized health claims have led to a growing interest in incorporating polyphenols as functional ingredients in food systems [3]. Nevertheless, the polyphenol-fortification of food matrices remains a major challenge given their inherent instability and susceptibility to degradation under environmental stresses, including exposure to light, oxygen, elevated temperatures, and fluctuations in pH [4]. In addition, most polyphenols have poor water solubility and are unstable under the alkaline conditions of the small intestine, which significantly reduces their bioaccessibility (fraction available for absorption after intestinal digestion) when orally consumed [1,5].

To overcome these challenges, various food-grade systems for encapsulation and delivery of polyphenols have been explored, including lipid carriers (emulsions, liposomes, solid lipid nanoparticles, oleogels), biopolymeric structures (gels/films/coatings/beads, nano/micro particles, conjugates, coacervates), surfactant assemblies (micelles, reverse micelles), and mixed systems (emulsion gels, Pickering emulsions, bigels) [5,6]. Among these, multiple emulsions (MEs)—particularly water-in-oil-in-water (W/O/W) systems—have attracted considerable interest as encapsulation systems for bioactive molecules. Structurally, they consist of a primary water-in-oil emulsion that is subsequently dispersed within an external aqueous phase, enabling the entrapment of compounds with both hydrophilic and lipophilic character. Consequently, this type of emulsions has been explored as a co-encapsulation strategy for a wide range of functional compounds, such as polyphenols, vitamins and carotenoids (catechin/curcumin, [7]; epigallocatechin gallate/lycopene, [8]; vitamin C/xanthoxylin, [9]; phycocyanin/astaxanthin, [10]; riboflavin/β-carotene, [11]; vitamin D3 and B9, [12]; vitamin B12 and D3, [13]). However, this complex structure also makes W/O/W emulsions more prone to destabilization than simple emulsions. Their destabilization can occur through different mechanisms [14], which become particularly critical when these systems are intended for oral delivery, since during gastrointestinal digestion they are exposed to changing pH conditions, mechanical stress, osmotic imbalances, and the action of enzymes and surfactants.

To reduce the instability associated with W/O/W emulsions—which limits their use as carriers for bioactive compounds in food systems—a number of stabilization approaches have been proposed, including proper selection of emulsifiers, adjustment of osmotic gradients, and stabilizing the interfaces with solid colloidal particles [6,7,15,16]. However, despite all these efforts, the effective retention of hydrophilic molecules within the inner aqueous compartment (W_1_) during long-term storage or gastrointestinal digestion is not consistently ensured, since the physicochemical characteristics of the encapsulated compound strongly influence this outcome [15,16]. In this context, spray drying provides an efficient approach for stabilizing W/O/W emulsions, since converting the liquid emulsion into dry powders embeds the droplets in a solid matrix, thereby hindering their coalescence and reducing the leakage of encapsulated compounds [17,18]. It represents the predominant technique for microencapsulating bioactive compounds in food applications, owing to its high process efficiency, versatility in formulation and processing, and suitability for large-scale [19]. Spray drying has been widely applied to enhance the stability of lipid-based delivery systems, including emulsions with bioactive compounds, liposomes, solid nanoparticles and nanostructured carriers [20]. However, its use in multicompartmental systems, particularly W/O/W emulsions, remains relatively limited. Although some studies have demonstrated its potential to enhance the long-term stability of these MEs [12,13,17,18,21,22], most research has focused on MEs designed as delivery systems for hydrophilic bioactives, with limited attention to co-encapsulation strategies that also incorporate lipophilic compounds [12,13,23]. Moreover, beyond the evaluation of physicochemical properties and storage stability of the resulting microparticles, few studies have assessed their performance under simulated gastrointestinal conditions, an important aspect for understanding their functionality as delivery vehicles. Notably, He et al. [13] and Ramzan et al. [12] evaluated the bioaccessibility of hydro- and liposoluble vitamins encapsulated in spray-dried MEs formulated with sodium caseinate–maltodextrin–sodium alginate and whey protein isolate–modified starch–carboxymethylcellulose blends, respectively, reporting high bioaccessibility values for both types of vitamins.

Different biopolymers have been employed as hydrophilic emulsifiers and encapsulating agents for the stabilization and spray drying of W/O/W emulsions. These include proteins [24], as well as blends composed of proteins and polysaccharides [12,13,25]. Hydrophobically modified starches, such as those chemically esterified with n-octenyl succinic anhydride, exhibit high surface activity and low viscosity. These properties enable high oil loading, promote the formation of stable emulsions, enhance oil retention, and allow for high encapsulation efficiency [19]. For these reasons, they have been widely used in the spray drying of oil-based flavors, micronutrients, and pharmaceuticals.

The present work focused on producing spray-dried microparticles from linseed oil (LO) MEs, formulated as co-delivery systems for chlorogenic acid (CGA) and curcumin (CU), using an octenyl succinic anhydride-modified starch derived from waxy maize (Capsul^®^, Grupo Mathiasen, Santiago, Chile) as the encapsulating agent, and to assess the influence of spray drying on the bioaccessibility of both bioactives and the major LO fatty acids under in vitro gastrointestinal conditions, compared to liquid multiple emulsions. Furthermore, their morphology, encapsulation efficiency, moisture content, particle size, and oxidative stability were also evaluated.

## 2. Materials and Methods

### 2.1. Materials

LO was purchased from Nutra Andes Ltd. (Valparaíso, Chile). Capsul^®^ was purchased from Grupo Mathiasen (Santigao, Chile). Sodium caseinate (NaCas) and polyglycerol polyricinoleate (PGPR) were sourced from Prinal S.A. and Dimerco S.A. (Santiago, Chile), respectively. Curcumin (CU) and chlorogenic acid (CGA) were purchased from Xi’an Xin Sheng Bio-Chem Co. (Xi’an, China) and AK Scientific (Union City, CA, USA), respectively. Porcine gastric mucosa pepsin (9001-75-6), porcine pancreatin (8049-47-6) and porcine bile extract (8008-63-7) were obtained from Sigma-Aldrich (Santiago, Chile).

### 2.2. Preparation of Multiple Emulsions (MEs)

Multiple Emulsions (MEs) were obtained by a two-step emulsification process (Figure S1) [26]. First, coarse W_1_/O emulsions were obtained by gradually incorporating the internal aqueous phase W_1_ (20%) into the oil phase (80%), composed of LO (94%) and PGPR (6%, *w*/*w*), using a mixer (TM31 Thermomix, Vorwerk, Wuppertal, Germany) operating at 3250 rpm and 60 °C for 15 min. The resulting coarse emulsion was then subjected to two passes in a two-stage high-pressure homogenizer (Panda Plus 2000, GEA, Parma, Italy), applying 550 bar in the first stage and 70 bar in the second. Subsequently, the fine W_1_/O emulsion (40%) was dispersed into the external aqueous phase W_2_ (60%) containing NaCas (0.5% *w*/*w*), using the same mixer at 700 rpm and 37 °C for 10 min to generate the coarse W_1_/O/W_2_ emulsion. This dispersion was further processed by two additional cycles of high-pressure homogenization (100/30 bar), yielding the final W_1_/O/W_2_ emulsion. This formulation, referred to as ME-C, did not contain bioactive compounds. Conversely, MEs with CGA (ME-CGA), CU (ME-CU), or both (ME-CGA/CU) were prepared by dissolving CGA in the W_1_ phase (1 mg/g) and CU in the oil phase (3 mg/g), achieving final concentrations of 80 ppm for CGA and 960 ppm for CU. The osmotic balance between W_1_ and W_2_ was adjusted with NaCl and verified with a 3320 osmometer from Advanced Instruments (Norwood, MA, USA) reduce diffusion effects. Table S2 summarizes the composition of the different formulations.

### 2.3. Characterization of MEs

#### 2.3.1. Oil Droplet Size and Size Distribution

Droplet dimensions of the emulsions, expressed as volume-weighted mean diameter (D_4,3_), together with their size distribution, were assessed through laser light scattering in a Horiba particle size analyzer (model LA-960, Kyoto, Japan; 650 nm) [16]. All measurements were carried out in triplicate at room temperature.

#### 2.3.2. Encapsulation Efficiency

Encapsulation efficiency was determined after centrifuging the MEs at 2400× *g* for 15 min to isolate the W_2_. Quantification of compounds in W_2_ was performed with a UPLC system (UltiMate 3000, Thermo Scientific, Waltham, MA, USA) equipped with a UV/VIS detector (VWD-3100, Thermo Scientific, Waltham, MA, USA) CGA was identified and quantified according to Qi et al. [27], employing a Symmetry C18 column (4.6 × 250 mm, 5 μm; Waters, Milford, MA, USA) and a calibration curve prepared from CGA standards (0.1–100 μg/mL; R^2^ = 0.99). For CU analysis, the method of Marczylo et al. [28] was applied, using an Acquity BEH Shield RP18 column (2.1 × 100 mm, 1.7 μm; Waters, Milford, MA, USA). CU quantification was carried out using a standard calibration curve within the same range (0.1–100 μg/mL; R^2^ = 0.99). Equation (1) was applied to determine the EE of CGA and CU:

(1) $$EE\;\left(\%\right)=100-\;\frac{B_{W2}\;\times 100}{B_{t0}},$$

where B_W2_ is the CGA or CU concentration in W_2_ after MEs preparation, B_t0_ is the total concentration of CGA or CU added to W_1_ or oil phase, respectively.

#### 2.3.3. Microstructure

The microstructure of MEs was assessed immediately after preparation, using an optical microscope (DM500, Leica Microsystems, Heerbrugg, Switzerland), coupled to a digital camera (Flexacam, Leica Microsystems, Heerbrugg, Switzerland), at 40 and 100 magnifications.

### 2.4. Formulation of Microparticle Systems

The formulation of spray-dried MEs without bioactive compounds was optimized using a central composite design (CCD) with star points (12 assays in total: 9 factorial points, 3 central replicates). Two factors were evaluated as independent variables: inlet air temperature (120–180 °C) and oil:Capsul^®^ ratio (1:3.3–1:6). The response variable was the EE of LO. To minimize bias, experiments were performed in randomized order. The experimental results were described by fitting a quadratic polynomial model, expressed in Equation (2):

(2) $$Y=\beta_{0}+\beta_{1}X_{1}+\beta_{2}X_{2}+\beta_{11}X_{1}^{2}+\beta_{22}X_{2}^{2}+\beta_{12}X_{1}X_{2}+\;\varepsilon ,$$

where the regression model coefficients (β_0_, β_i_, β_ii_, and β_ij_) represented the intercept, linear, quadratic, and interaction terms, respectively, and ε corresponded to the residual error.

The optimum values of the independent factors for maximizing EE were estimated using response surface methodology (RSM). Data analysis included ANOVA, lack-of-fit evaluation, coefficient estimation, and 3D surface plot visualization, which were performed with Statgraphics software (software 6.0, Manugistics Inc., Rockville, MA, USA).

For spray drying, the infeed dispersion was prepared by mixing an aliquot of MEs (10.93 g) with a Capsul^®^ dispersion (11.53–20.98 g) in water (77.54–68.09 g) using an orbital shaker (JSSI-100C, JSR, Yongin, Republic of Korea) at 200 rpm for 15 min. The mixture was subsequently introduced into a mini spray dryer (B-290, Büchi, Flawil, Switzerland), a co-current system in which the atomized droplets and the hot air stream moved in the same direction through the drying chamber. Atomization occurred at the dual-fluid nozzle (0.7 mm diameter), which dispersed the liquid feed into fine droplets. The equipment was equipped with an inlet air heater set at the operating temperature, a drying chamber where heat transfer took place between the air and the droplets, and a cyclone separator that collected the dried powder from the air stream. Drying was with an air flow of 600 L/h, a feed rate of 2 mL/min, an atomization pressure of 20 psi, and inlet air temperatures between 120 and 180 °C. The resulting MPs were kept at −20 °C until further analysis. Spray drying of MEs containing bioactive compounds (ME-CGA, ME-CU, ME-CGA/CU) was performed using the inlet air temperature and oil:Capsul^®^ ratio identified as optimal by the statistical design. As a result, four MPs systems were obtained: MP-C (without bioactives), MP-CGA (with CGA in W_1_), MP-CU (with CU in the oil phase) and MP-CGA/CU (with CGA in W_1_ and CU in the oil phase).

### 2.5. Characterization of Microparticle Systems

The microparticle systems prepared under optimal conditions, according to the experimental design, were evaluated with respect to the following characteristics.

#### 2.5.1. Encapsulation Efficiency of Linseed Oil

The quantification of surface oil of the MP-C, MP-CGA, MP-CU, and MP-CGA/CU formulations was performed according to Shamaei et al. [29]. In brief, 1 g of MPs was mixed with 10 mL of hexane in a pre-weighed flask, and the resulting suspension was filtered through Whatman No. 1 filter paper. The paper was rinsed three times with 10 mL of hexane. The solvent was subsequently removed by vacuum evaporation (R250, Büchi, Flawil, Switzerland). The amount of surface oil was determined from the difference in weight between the initial pre-weighed flask and the same flask after oil extraction. The EE of LO for the MP systems was determined using Equation (3):

(3) $$EE\;of\;LO\left(\%\right)=\frac{Total\;oil-Surface\;oil}{Total\;oil}\times 100,$$

where total oil corresponds to the theoretical total oil contained in the MPs, as LO is not volatile, and oil loss during spray drying is considered negligible.

#### 2.5.2. Encapsulation Efficiency of Chlorogenic Acid and Curcumin

To quantify the total CGA content in MP-CGA and MP-CGA/CU microparticles, 1 g of MPs was dispersed in 5 mL of Milli-Q water, vortexed (1 min) and sonicated (10 min). This cycle was repeated three times. Subsequently, 3 mL of hexane were added, and the mixture was vortexed again for 1 min and centrifuged at 9056× *g* for 5 min at 4 °C. The aqueous phase was collected and filtered through a 0.22 μm membrane. Surface CGA content in MP-CGA and MP-CGA/CU was determined by dispersing 1 g of microparticles in 5 mL of methanol, followed by gentle manual agitation to ensure complete dispersion. The suspension was centrifuged at 123× *g* for 1 min at 4 °C, and 3.5 mL of the supernatant was collected. The solvent was eliminated using a rotary evaporator (R-100, Büchi, Flawil, Switzerland), and the dry extract was redissolved in Milli-Q water (1 mL) before being passed through a 0.22 μm membrane filter for analysis. For both total and surface CGA samples, identification and quantification were performed as detailed in Section 2.3.2.

Surface CU content in MP-CU and MP-CGA/CU was determined by dispersing 100 mg of MPs in 4 mL of isopropanol by gentle manual shaking. After filtration through Whatman No. 1 paper, the CU content was quantified by UPLC (Section 2.3.2). The total CU content corresponded to the amount initially incorporated into the MPs.

Equation (4) was applied to determine the EE of CGA and CU in the MPs:

(4) $$EE\left(\%\right)=\frac{Total\;CGA\;or\;CU\;-\;Surface\;CGA\;or\;CU}{Total\;CGA\;or\;CU}\times 100.$$

#### 2.5.3. Oxidative Stability

To evaluate oxidative stability, MPs were analyzed in a Rancimat system (model 892, Metrohm Ltd., Herisau, Switzerland), and the induction period (IP) was recorded. This parameter reflects the time until the onset of secondary lipid oxidation products. Microparticle systems prepared under optimal conditions (MP-C, MP-CGA, MP-CU, and MP-CGA/CU) were tested at 80 °C under a constant airflow of 20 L/h. For comparison, the IP of non-encapsulated LO was also measured.

#### 2.5.4. Particle Size and Morphology

The MPs produced under optimized conditions were evaluated in terms of particle size and morphological characteristics. Particle size was determined by laser diffraction using a Horiba LA-960 (Kyoto, Japan) equipped with a 300 mm lens. The samples were dispersed in propylene glycol, and the results were reported as D_4,3_ values. Morphological features were evaluated by scanning electron microscopy (SEM; LEO 1420VP, LEO Electron Microscopy Ltd., Cambridge, UK) operated at 20 kV. Prior to imaging, the samples were coated with a thin layer of gold/palladium using a vacuum evaporator (PS 10E, Varian, Grove City, OH, USA).

#### 2.5.5. Moisture Content

An infrared balance (Radwag PMR 50/1/NH, Radom, Poland) was employed to quantify the moisture content of MP-C, MP-CGA, MP-CU, and MP-CGA/CU.

#### 2.5.6. Droplet Size and Size Distribution of Rehydrated Microparticles

The microstructure of the recovered ME-CGA/CU, obtained by rehydrating MP-CGA/CU, was assessed as described in Section 2.3.3. Based on Equation (5), droplet size and distribution were obtained from five images at 40× magnification, using measurements of 300 droplets processed with Levenhuk ToupView software (v4.11.19728.20211022, Hangzhou, China).

(5) $$D_{4,3}=\sum n_{i}d_{i}^{4}/\sum n_{i}d_{i}^{3},$$

with *n_i_* representing the number of droplets of diameter *d_i_*.

### 2.6. Evaluation of Multiple Emulsions and Spray-Dried Multiple Emulsions Under Simulated Gastrointestinal Digestion Conditions

In vitro gastrointestinal digestion of ME-CGA/CU and MP-CGA/CU was performed, reproducing human oral, gastric, and intestinal stages. The simulation of the process and preparation of the corresponding digestive fluids were carried out according to the INFOGEST protocol [30]. The oral, gastric, and intestinal phases were conducted at pH 7.0 for 2 min, pH 2.0 for 2 h, and pH 7.0 for 2 h, respectively. All stages were performed at 37 °C under continuous shaking. The amounts of CGA and CU released after each stage of digestion were quantified by UPLC as detailed in Section 2.3.2. To determine the bioaccesible fraction of CGA and CU, the aqueous phase containing the micellar fraction was collected after the intestinal stage by centrifuging the digested samples at 9056× *g* for 60 min at 4 °C.

The fatty acids released during digestion were extracted from the micellar phase, methylated into fatty acid methyl esters, and subsequently identified and quantified by gas chromatography (7890B, Agilent Technologies, Santa Clara, CA, USA) [16]. Quantification of oleic (C18:1), linoleic (C18:2), and α-linolenic (C18:3) acid methyl esters was based on calibration curves (R^2^ = 0.99).

Equation (6) was applied to calculate the bioaccessibility of CGA, CU and major fatty acids after the intestinal stage:

(6) $$Bioaccessibility\left(\%\right)=\left(\frac{CGA\;or\;CU\;or\;fatty\;acid\;in\;micellar\;phase}{CGA\;or\;CU\;or\;fatty\;acid\;in\;non\;-\;digested\;sample}\right)\times 100.$$

### 2.7. Statistical Analysis

The data were analyzed with Statgraphics Centurion 15.1 (StatTechnologies Inc., The Plains, VA, USA) using ANOVA and Tukey’s post hoc test to determine significant differences at *p* ≤ 0.05.

## 3. Results

### 3.1. Microstructure, Droplet Size, and Encapsulation Efficiency of Multiple Emulsions

**Figure 1:**
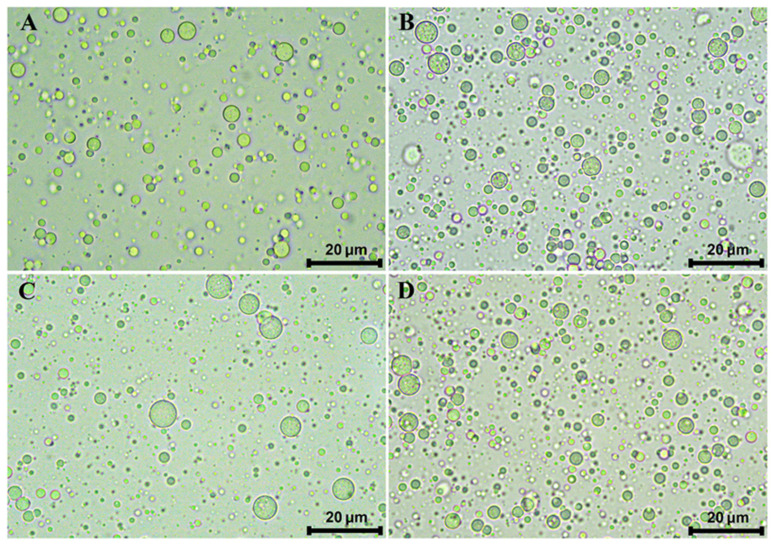
Micrographs of the MEs obtained with an optical microscope at 100×. (**A**): ME-C; (**B**): ME-CGA; (**C**): ME-CU; (**D**): ME-CGA/CU.

As depicted in Figure 1, ME-C, ME-CGA, ME-CU, and ME-CGA/CU exhibited the typical multi-compartment structure of MEs, with oil droplets containing smaller internal water droplets. Regarding oil droplet size, expressed as D_4,3_ values (Table 1), all MEs presented values of approximately 2 µm and displayed a monomodal size distribution (Figure S2A), indicating that the incorporation of bioactive compounds did not affect droplet size.

Monomodal and bimodal size distributions have been reported for similar MEs stabilized with NaCas and PGPR as hydrophilic and lipophilic emulsifiers, respectively [26]. The size and distribution of W_1_/O droplets can be affected by various factors, including processing conditions, interfacial properties, nature and concentration of emulsifiers, oil phase composition, phase volume fractions and the viscosity of each phase [14], making comparisons across studies difficult. The shear forces applied during emulsification, together with the overall energy input, are key factors influencing droplet size and emulsifier efficiency in MEs stabilized with PGPR and NaCas. For instance, when only a magnetic stirrer was used to prepare stable olive oil MEs (D_4,3_ 8.6–15.9 μm), approximately 12.5% *w*/*w* of NaCas was required [31]. Similarly, using a Couette cell, more than 6% NaCas was needed to obtain sunflower MEs with D_4,3_ values around 6 μm [32]. However, when similar ME systems were prepared by high-pressure homogenization, applying higher shear forces and energy, much lower amounts of NaCas (0.5% *w*/*w*) were sufficient to produce vegetable oil MEs with small droplets of 1.5 and 2.5 μm [26].

The EE of CGA in all MEs was around 60% (Table 1), with no significant differences between the system encapsulating only CGA and that co-encapsulating CU in the oil phase (*p* > 0.05). Dima & Dima [33] reported higher efficiencies (~80%) in LO MEs stabilized with a Span 80-lecithin blend at the internal interface and Tween 20 at the external interface. The lower EE values found in this work could result from employing different emulsifiers (PGPR and NaCas). In any case, CGA exhibited low encapsulation stability, with 90% of the compound being released within the first four days of storage [33]. Therefore, spray drying of MEs could be a suitable strategy to encapsulate and immobilize CGA in the W_1_ phase.

In contrast to the lower EE values of CGA, CU showed efficiencies close to 100%, which were not affected by the presence of CGA, as similar values were obtained in MEs containing only CU and in those also co-encapsulating CGA. Aditya et al. [7] co-encapsulated CU and catechin in the oil phase and W_1_, respectively, using PGPR and Tween 80 as emulsifiers in olive oil-based MEs. They reported lower EE values for CU (~88%) and found no significant differences compared to systems where only CU was encapsulated. Similarly, Paredes-Toledo et al. [16] studied the impact of co-encapsulating CGA and CU on the encapsulation efficiency of both compounds in MEs with the same composition as those used in this study but with larger oil droplet sizes. CGA and CU showed similar EE values under single and co-loading conditions. This was mainly attributed to their localization in distinct compartments within the ME structure, driven by their differing hydrophobicity, which limits potential interactions.

### 3.2. Formulation of Microparticle Systems

The experimental conditions used for the formulation of MPs, along with the corresponding EE of LO are presented in Table 2. The EE of LO ranged from 86.6% to 95.0%. Statistical analysis indicated that both the linear effect of the oil:Capsul^®^ ratio and its interaction with inlet air temperature significantly influenced the response (*p* ≤ 0.05), whereas only the quadratic component of inlet air temperature showed a significant contribution (*p* ≤ 0.05). ANOVA results (Table S1), revealed that the model accounted for 89.8% of the variability in EE of LO data (R^2^ adjusted for degrees of freedom), with residuals below 2.0 and no significant lack-of-fit (*p* > 0.05).

**Table 2:** Experimental design for microparticle formulation.

Run	LO:Capsul^®^ Ratio	Inlet Air Temperature (°C)	EE of LO (%)
1	1:3.02	150	87.2 ± 0.38
2	1:6.28	150	93.6 ± 0.17
3	1:6	180	94.0 ± 0.80
4	1:3.3	180	91.8 ± 0.79
5	1:4.65	113.7	93.9 ± 0.77
6	1:3.3	120	86.6 ± 0.05
7	1:6	120	94.9 ± 0.92
8	1:4.65	186.3	95.0 ± 0.02
9	1:4.65	150	89.8 ± 0.32
10	1:4.65	150	89.3 ± 0.13
11	1:4.65	150	89.7 ± 0.66
12	1:4.65	150	91.2 ± 0.28

EE: Encapsulation efficiency; LO: Linseed oil.

**Figure 2:**
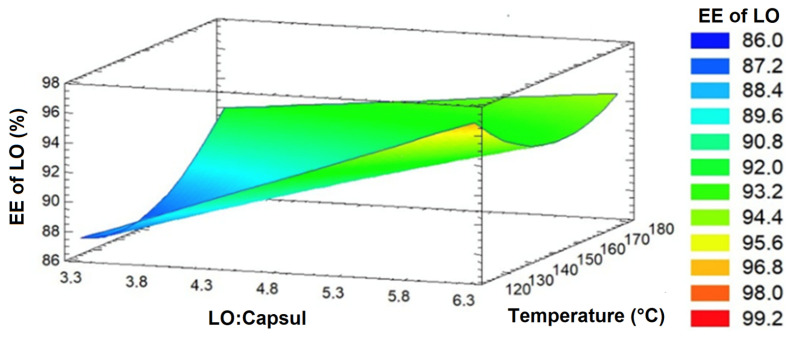
Response surface graph for the EE of LO in MP.

The response surface plot (Figure 2) shows that higher Capsul^®^ content led to increased EE of LO at both low and high temperatures. In the spray-drying microencapsulation of polyunsaturated oils—such as LO—it is desirable to obtain microparticles with high EE, since this reduces the amount of free surface oil, thereby lowering susceptibility to oxidation and enhancing oxidative stability. The proportion of coating material relative to the core oil is an important variable affecting the EE of LO [34], and higher solids content has been consistently associated with both increased EE of LO and reduced surface oil in spray-dried microparticles, as a sufficient amount of encapsulating agent is necessary to effectively coat and encapsulate the oil droplets [34].

In addition, Figure 2 shows that the inlet air temperature also influenced the EE, although to a lesser extent than the solids content. At high oil:Capsul^®^ ratios—conditions that yielded the highest EE—the EE of LO tended to be higher at both low and high inlet air temperatures. Drying conditions, particularly the inlet air temperature, influence several parameters of the resulting microparticles, including morphology, encapsulation efficiency, and the retention of the encapsulated compounds [34]. However, in the majority of cases, studies lack an explanation for the inlet air temperature chosen for spray drying.

According to the model, the maximum EE of LO (98%) was obtained at an oil:Capsul^®^ ratio of 1:6.28 and an inlet air temperature of 114 °C, conditions that fell within the experimental range. Such drying conditions are consistent with those typically applied for oils rich in polyunsaturated fatty acids: inlet temperatures are generally reported between 110 and 220 °C, while oil-to-encapsulating agent ratios usually range from 1:1 to 1:10 [35].

### 3.3. Characterization of Microparticle Systems

Table 3 shows the total LO content, the EE of LO, the induction period (IP), the moisture content, the EE of CGA and CU, and the size (D_4,3_ values) of the microparticles prepared under optimal conditions (oil:Capsul^®^ ratio 1:6.28; inlet air temperature 114 °C) from the MEs.

#### 3.3.1. Encapsulation Efficiency of Linseed Oil

The total LO content was similar in all the microparticle systems, with values around 136 mg/g (Table 3). The EE of LO exceeded 90% in all cases, which is considered adequate for oil encapsulation by spray drying [35,36]. As previously noted, the EE of oil is a key parameter in spray-drying encapsulation, as it is generally associated with enhanced oxidative stability due to the reduced surface oil exposed to environmental conditions [35].

This parameter is influenced by multiple factors, including total solids content and its ratio to the oil phase, the drying conditions—particularly inlet air temperature—type of encapsulating agent, emulsification method, and properties of the resulting emulsion [35]. Concerning the latter, evidence indicates that when oil-in-water emulsions are produced using high-pressure homogenization, the resulting microparticles exhibit reduced surface oil content due to smaller droplet sizes [34,36]. This may partly account for the high EE of LO observed in this study, as the MEs were prepared using high-pressure homogenization at 100 bar during the second emulsification step, yielding droplet sizes of approximately 2 µm.

Previous studies have reported similar EE values for LO using different carbohydrate-based encapsulating agents. However, in those cases, the emulsions subjected to spray drying were simple oil-in-water systems rather than MEs. Domian et al. [37] reported EE values ranging from 95% to 99% when modified tapioca starch–trehalose blends were employed as encapsulating agents. Gallardo et al. [36] obtained values close to 90% with gum arabic and blends of gum arabic with maltodextrin and whey protein isolate. In contrast, when methylcellulose was combined with maltodextrin, the EE of LO dropped significantly to 25%, highlighting the critical role of the encapsulating agent in retaining and protecting the encapsulated oil. Similarly, high EE values for LO (80–90%) have been reported using protein-based encapsulating agents such as intact and hydrolyzed rice protein [38], sodium caseinate, or whey protein concentrate [39]. EE values of LO were similar among the different systems, regardless of the incorporation of CGA and/or CU into the MEs (Table 3), as both MP-CGA and MP-CU exhibited comparable values to MP-C.

#### 3.3.2. Oxidative Stability

The IP values obtained for each microparticle formulation are presented in Table 3. For comparison, the IP of non-encapsulated LO was also determined, yielding a value of 19.4 ± 1.3 h. This result is consistent with previous studies reporting similar IP values for LO (17.4 h; [40]). The relatively high susceptibility of LO to oxidation is attributed to the high proportion (approximately 50–60%) of α-linolenic acid [39]. Despite this susceptibility, non-encapsulated oil exhibited a significantly longer IP (*p* ≤ 0.05) than the control microparticles (MP-C), which showed an IP of 10.2 ± 1.4 h. This result aligns with previous reports showing lower oxidative stability in microencapsulated oils compared to bulk oils [35], likely due to oxygen incorporation and increased surface area during emulsification previous spray drying process [41].

The incorporation of CGA in W_1_ and/or CU in the oil phase of MEs increased (*p* ≤ 0.05) the IP values of the microparticles (MP-CGA/CU ~20–24 h) compared to MP-C (~10 h), thereby improving the oxidative stability. The addition of antioxidant compounds to spray-dried microencapsulated polyunsaturated oils is a widely explored strategy to enhance oxidative stability [42]. Both natural extracts (such as rosemary, sage or myrtle leaf extract) and pure antioxidant compounds (such as ascorbic acid, hydroxytyrosol and hydroxytyrosol alkyl esters, or blends of lipophilic eugenol and β-carotene) have been incorporated into feed emulsions with the aim of reducing oxidation and improving oil stability [35,41,42]. As shown in Table 3, CU addition led to a slightly greater increase in IP values than CGA (*p* < 0.05) although CU was present at a much higher concentration than CGA in MP (412 ppm vs. 34 ppm). This finding may be explained by differences in the antioxidant efficacy between CGA and CU. One parameter commonly used to compare the antioxidant capacity of different compounds is their oxidation potential, as it reflects their ability to donate electrons. In this context, CGA exhibits a lower oxidation potential (0.26–0.31 V; [43,44]) than CU (0.66 V; [45]), suggesting a greater electron-transferring ability and higher antioxidant efficacy under these conditions. When both bioactive compounds were incorporated simultaneously, the IP did not exceed the values observed for the individual compounds, suggesting the absence of a synergistic antioxidant effect. This contrasts with previous reports describing synergistic antioxidant activity between CU and water-soluble antioxidants such as catechin or epigallocatechin gallate, which are capable of regenerating CU radicals due to their lower oxidation potential [46].

#### 3.3.3. Particle Size and Morphology

**Figure 3:**
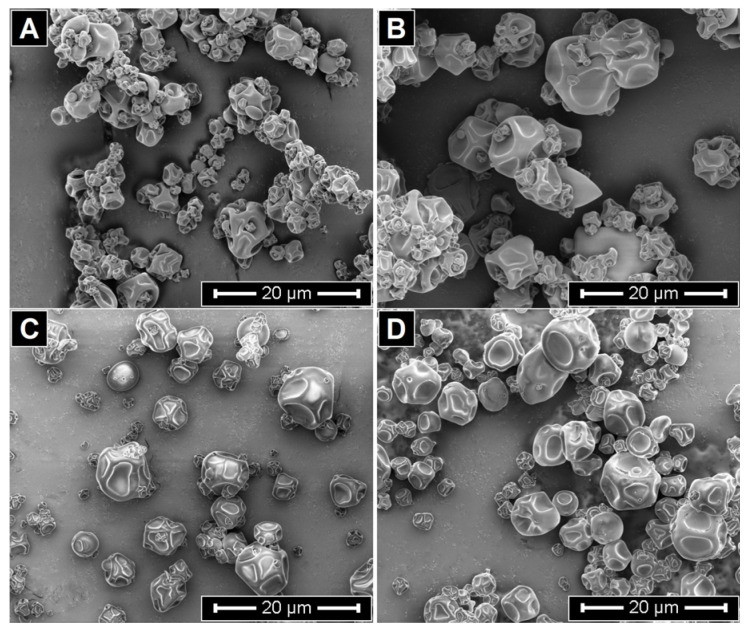
SEM Micrographs of MPs. MP-C (**A**), MP-CGA (**B**), MP-CU (**C**), and MP-CGA/CU (**D**).

The D_4,3_ values of the microparticle systems are presented in Table 3. The microparticles exhibited D_4,3_ values ranging from 7 to 13 µm, consistent with previous reports for spray-dried LO microparticles prepared using various encapsulating agents [37,39]. These results are also in line with the particle size distribution, in which most particles ranged between 2 and 20 µm (Figure S2B). With respect to the morphology of the MPs (Figure 3 and Figure S3), SEM images revealed particles that were irregular in shape, approximately spherical, with multiple surface indentations, a tendency to agglomerate, and a marked size polydispersity. This morphology has been previously described in oil microparticles encapsulated with modified starch [41,47]. Surface shrinkage in spray-dried microparticles is typically associated with the initial phase of drying and may develop under both high and low inlet temperatures [48]. In the present work, this phenomenon was detected at 114 °C, which is attributed to the reduced rate of water diffusion, providing additional time for particle contraction during the formation of the outer layer [41]. Images of the spray-dried powders are shown in Figure S4.

#### 3.3.4. Moisture Content

The moisture content of the MP systems is shown in Table 3. This parameter is critical in the shelf life of the powders, as it directly affects their stability. The microparticles exhibited moisture contents varied between 3.7% and 4.9%. Despite statistically significant differences (*p* ≤ 0.05) among samples, the values remained within the expected range for spray-dried products and were below 5%, ensuring the microbiological stability of the microparticles [42].

#### 3.3.5. Encapsulation Efficiency of Chlorogenic Acid and Curcumin

Encapsulation efficiencies for CGA and CU in microparticles are summarized in Table 3. When encapsulated separately in MP-CGA and MP-CU formulations, both compounds exhibited EE of approximately 90%. Upon co-encapsulation, similar values were obtained (87.8 ± 1.5% for CGA and 88.9 ± 0.6% for CU), with no significant differences (*p* > 0.05) due to co-encapsulation. This suggests that the presence of one bioactive compound does not interfere with the encapsulation efficiency of the other, possibly due to their localization in different compartments within the ME. Compared to the efficiencies observed for CGA in ME systems, spray drying significantly increased the EE of CGA by approximately 30% (from 60.1 ± 0.3% to ~87–90%), both when encapsulated alone and when co-encapsulated with CU. This enhancement may be explained by the starch crust formed around the oil droplets, which could entrap CGA previously released into the W_2_ phase in ME. The encapsulation of CGA by spray drying—often from coffee or coffee wastes and employing different encapsulating agents—has been documented in several studies [49,50,51]. Encapsulation efficiencies similar to those obtained in this study (close to 90%) have been achieved using maltodextrin [50] or a mixture of fructans and gum arabic [51].

In contrast to the EE of CGA, the spray drying process slightly reduced the EE of CU compared to that in ME, decreasing from 99% to approximately 90%. This reduction could be explained by the occurrence of non-encapsulated oil in the MP systems, where CU is dissolved. In spite of this reduction, EE values around 90% are still high and exceed those reported for other lipophilic bioactives such as vitamin D_3_ encapsulated in the oil phase of spray-dried MEs (62–68% using a blend of sodium caseinate, maltodextrin and polyssacharides at an oil:encapsulating agent ratio of 1:2; [13]). These results can be explained by the use of a low oil:encapsulating agent ratio (1:6.28), determined through experimental design as optimal for maximizing the EE of LO. As previously discussed, this low ratio is a key factor contributing to high oil retention and, consequently, to the EE of CU dissolved in the oil phase. Several studies have encapsulated CU by spray drying, typically dissolving the compound in ethanol and mixing it with the encapsulating agent dispersion. For example, Guo et al. [52] reported a CU EE of 82.5% using an inulin–maltodextrin–tamarind gum blend at a high inlet temperature (190 °C). In contrast, other combinations of encapsulating agents led to lower EE values, ranging from 41% to 78%. In our study, the CU EE exceeded those reported by Guo et al. [52]. This difference can be attributed not only to differences in the encapsulating agents used, but also to the method of CU incorporation into the infeed dispersion, as its dissolution in the oil phase of the ME may have enhanced its retention during the drying process thanks to the higher boiling point of oil compared to organic solvents.

#### 3.3.6. Droplet Size and Size Distribution of Rehydrated Microparticles

**Figure 4:**
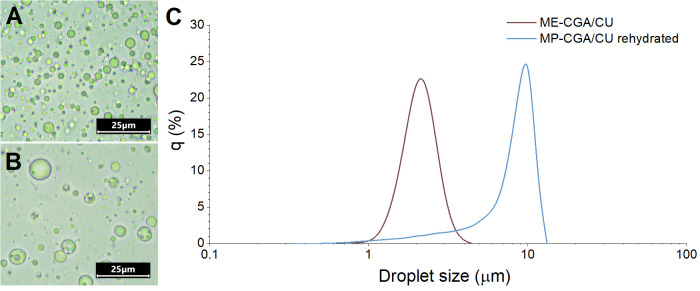
Microstructure of ME-CGA/CU (**A**), and rehydrated MP-CGA/CU (**B**), droplet size distribution of ME-CGA/CU and rehydrated MP-CGA/CU (**C**).

From the four microparticle systems initially prepared (MP-C, MP-CGA, MP-CU and MP-CGA/CU) from the corresponding multiple emulsions (ME-C, ME-CGA, ME-CU, and ME-CGA/CU), the MP-CGA/CU system was selected for detailed evaluation of rehydration behavior and in vitro release during digestion, since it represents the co-delivery approach proposed as the main aim of this work. The rehydration effect of the microparticles is particularly relevant, as both their potential incorporation into aqueous foods and their digestion involve the rehydration, which could restore their original structure prior to drying. MP-CGA/CU microparticles recovered the typical microstructure of MEs upon rehydration in distilled water (Figure 4B), with inner water droplets visible inside the oil droplets. However, oil droplets were larger in rehydrated MP-CGA/CU (Figure 4B) than those in ME-CGA/CU (Figure 4A), indicating that the drying and/or the rehydration process promoted oil droplet coalescence. Furthermore, fewer and larger inner water droplets were also observed, suggesting partial leakage of W_1_ and coalescence of water droplets. This microstructural change could enhance CGA release upon rehydration of the microparticles during in vitro digestion. The increase in oil droplet size in rehydrated MP-CGA/CU was confirmed by the higher D_4,3_ values (6.9 ± 0.8 µm vs. 2.0 ± 0.0 µm in ME-CGA/CU) and by the shift in size distribution towards higher values (Figure 4C). Similar behavior has been reported for microparticles obtained from freeze-dried or spray-dried MEs and has similarly been attributed to oil droplet coalescence during drying, caused by the shear forces applied during atomization [12,13,24].

#### 3.3.7. Evaluation of Multiple Emulsions and Spray-Dried Multiple Emulsions Under Simulated Gastrointestinal Digestion Conditions

**Figure 5:**
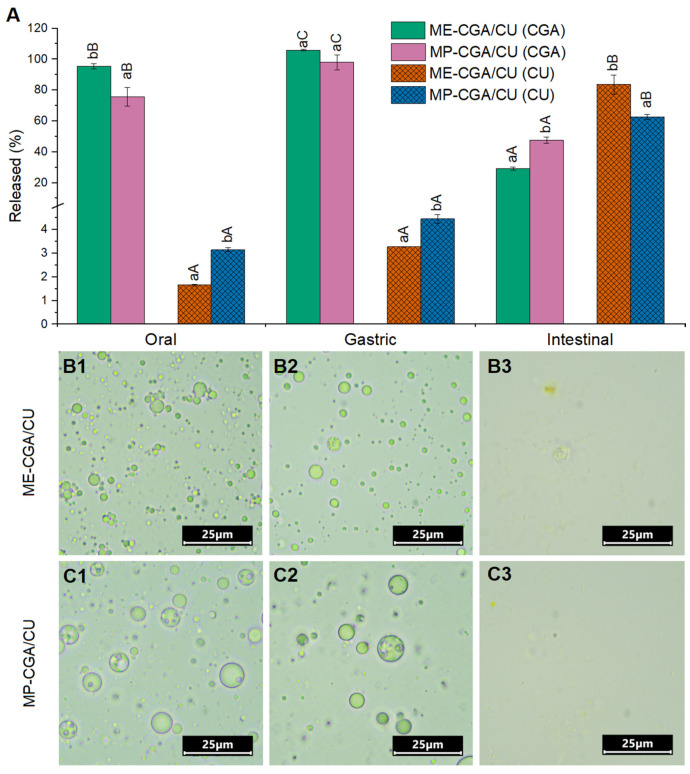
Release of chlorogenic acid (CGA) and curcumin (CU) from ME-CGA/CU and MP-CGA/CU after each digestion stage (**A**). Optical micrographs of MEs (**B**): ME-CGA/CU and (**C**): MP-CGA/CU after oral (1), gastric (2), and intestinal (3) stages. For each digestion stage, significant differences (*p* ≤ 0.05) between samples are denoted by distinct lowercase letters (a–b). Within a given sample, significant differences (*p* ≤ 0.05) in bioactive release across stages are denoted by capital letters (A–C).

Figure 5 shows the release behavior of CGA and CU from ME-CGA/CU and MP-CGA/CU, together with their microstructure at the three stages of the simulated gastrointestinal digestion.

During the oral stage of digestion, the total CGA released was 95.5 ± 1.7% and 75.7 ± 6.0% in ME-CGA/CU and MP-CGA/CU, respectively. After this stage, the oil droplets maintained their microstructure with internal water droplets in both systems (Figure 5(B1,C1), indicating that the main driver of CGA release during this phase was likely the osmotic gradient between W_1_ and SSF (8 and 39 mOsm/Kg, respectively). As shown in Figure 5(C1), the microparticles underwent complete disintegration during the oral phase, releasing the encapsulated ME. This behavior is most likely due to the rapid dissolution of the encapsulating agent in the simulated salivary fluid, rather than enzymatic hydrolysis of modified starch by α-amylase, given the very short contact time in this digestion phase (only two minutes). In fact, OSA starch has been reported to be highly resistant to enzymatic hydrolysis, as this chemical modification impairs enzyme binding to starch [53]. Nevertheless, the amount of CGA released from MP-CGA/CU was lower than that from ME-CGA/CU, suggesting that, although OSA starch dissolves in the simulated salivary fluid (Figure 5(C1)), it may form a network within the digestion fluid that restricts CGA molecular diffusion and slows its release. Furthermore, OSA starch may reinforce the external interface of the ME given its emulsifying capacity [53] and its ability to establish hydrogen bonding and electrostatic interactions with NaCas [54,55], which could further limit CGA release.

After the gastric phase, complete release of CGA was observed in both systems. Although W_1_/O droplets retaining the characteristic multicompartmentalized structure were still visible (Figure 5(B2,C2)), a higher number of empty droplets was observed in the ME-CGA/CU compared with MP-CGA/CU, suggesting a stabilizing role of Capsul^®^ at the external interface of MEs, as this modified starch resists pepsinolysis during gastric conditions. In spite of this, MP-CGA/CU showed a slight enlargement of oil droplets after the gastric phase, suggesting some coalescence. In this context, Lin et al. [53] reported that OSA starch can stabilize O/W emulsions during the gastric phase mainly by steric hindrance, as the carboxyl groups of OSA starch are mostly protonated at this pH. However, OSA starches with low degree of substitution as Capsul^®^ (0.0223 ± 0.0018; [56]) led to a higher oil droplet coalescence as observed in this study. At this stage, bioactive compounds entrapped in the W_1_ phase of MEs may be released as a result of various factors, including the nature of the emulsifiers at the interfaces, the hydrophilicity of the encapsulated compound and the osmotic imbalance between W_1_ and SGF. The susceptibility of NaCas to pepsin-mediated hydrolysis likely contributed to the loss of internal droplets observed in both systems during gastric digestion due to the weakening of the external interface, especially in the case of ME-CGA/CU. In addition, the osmotic imbalance between the W_1_ phase of MEs and the surrounding aqueous media has been identified as a key driver of CGA release during digestion [15,16,57], as water diffuses across the interfaces in response to osmotic gradients. The highly hydrophilic nature of CGA facilitates this process, although the extent of release may be modulated by the interfacial composition. For example, when the internal interface of MEs is stabilized with hydrophobic nanoparticles, CGA diffusion is hindered because the hydrophobic barrier limits transport of hydrophilic compounds driven by osmotic pressure [16]. However, full gastric release of CGA was observed when the interfaces were stabilized by the emulsifiers PGPR and NaCas [16], as observed in ME-CGA/CU and rehydrated MP-CGA/CU in this study. In contrast, the encapsulation of less hydrophilic molecules in W_1_ of MEs leads to different release profiles. For example, Lee et al. [21] encapsulated peanut sprout extract, rich in resveratrol (~110 µg/g), in spray-dried MEs and observed minimal resveratrol release (<3%) after simulated gastric digestion, while achieving a bioaccessibility of approximately 70–80%. This behavior can be explained by the physicochemical properties of resveratrol, which is nearly ten times more soluble in triacylglycerol oils than in water [58], resulting in limited gastric release. Similarly, Hu et al. [24] reported approximately 90% release of vitamin C, a highly hydrophilic compound, from spray-dried MEs after the gastric phase, further illustrating the dominant role of compound hydrophilicity and osmotic forces in governing release behavior.

The bioaccessibility of CGA after the intestinal phase was 29.2 ± 0.9% for ME-CGA/CU and 47.6 ± 2.0% for MP-CGA/CU. Since CGA was completely released during the gastric phase in both systems, these results indicate that a substantial fraction of the compound degraded under intestinal conditions. The higher bioaccessibility observed in MP-CGA/CU suggests that OSA starch provided protection against intestinal degradation, likely through specific molecular interactions. In fact, CGA can interact with amylopectin in starch-based carriers via hydrogen bonding and CH–π interactions, which enhances its stability in alkaline environments [59]. Across the different digestive phases, the pH conditions to which bioactive compounds are exposed largely determine their stability and, consequently, their bioaccessibility. While CGA exhibits stability during the gastric stage, it becomes unstable once exposed to the slightly alkaline conditions of the intestine, where it may undergo isomerization and degradation, substantially lowering its bioaccessibility [59,60]. This behavior contrasts with other hydrophilic bioactive compounds encapsulated in MEs. In particular, He et al. [13] and Ramzan et al. [12] studied the encapsulation of vitamins B_12_ and B_9_ in spray-dried MEs stabilized with NaCas or whey protein isolate, using modified starch (15% w/w in W_2_; oil:modified starch ratio 1:1.5) as the encapsulating agent. In both studies, approximately 60% of the encapsulated vitamins were released after the gastric phase; however, bioaccessibility values approached 90%. Unlike CGA, vitamins B_12_ and B_9_ are mainly susceptible to degradation under prolonged exposure to highly acidic environments [12,13], which may explain their comparatively higher stability and bioaccessibility under intestinal conditions.

In the context of MEs, previous studies have reported higher bioaccessibility values for other water-soluble phenolic compounds. For instance, Aditya et al. [7] observed 54% bioaccessibility for catechin encapsulated in the W_1_ phase of MEs stabilized with Tween 80, a result attributed to the high EE of catechin (97%), the stability of Tween 80 under gastric conditions, and the protective effect of CU at slightly alkaline pH. Similarly, Paredes-Toledo et al. [16] reported ~60% CGA bioaccessibility in MEs stabilized with PGPR and NaCas at the internal and external interfaces, respectively. In that formulation, however, the external interface was further stabilized with pectin, and the oil droplet diameter (24.5 ± 0.1 µm) was more than ten times larger than that obtained in the present study (2.0 ± 0.0 µm).

Curcumin release after the gastric phase was less than 5% in both systems (Figure 5A), a significantly lower value than that observed for CGA at this stage, likely due to the hydrophobic nature of CU. However, a significant amount of CU was released during the intestinal phase, reaching bioaccessibility values of ~60–80%. Complete collapse of the oil droplets was observed after intestinal digestion in both systems (Figure 5(B3,C3)). This collapse resulted from the displacement of NaCas from the external interface by bile salts and lipases and/or hydrolysis by pancreatic enzymes in the simulated intestinal fluids, which exposed the oil phase to lipolysis catalyzed by pancreatic lipases. In rehydrated MP-CGA/CU, Capsul^®^—resistant to pepsin in the stomach—is further hydrolyzed by pancreatic amylase, contributing to the weakening the droplet interfaces [53]. As a consequence of lipolysis, mixed micelles are formed, which facilitates solubilization and enhances CU bioaccessibility. Consistently, Aditya et al. [7] reported high CU bioaccessibility (~72%) in olive oil-based MEs after simulated gastrointestinal digestion. In general, lipid-based delivery systems such as MEs enhance the bioaccessibility of hydrophobic compounds like CU because lipolysis in the intestine generates mixed micelles, aided by bile salts, that entrap these poorly water-soluble molecules [7].

**Figure 6:**
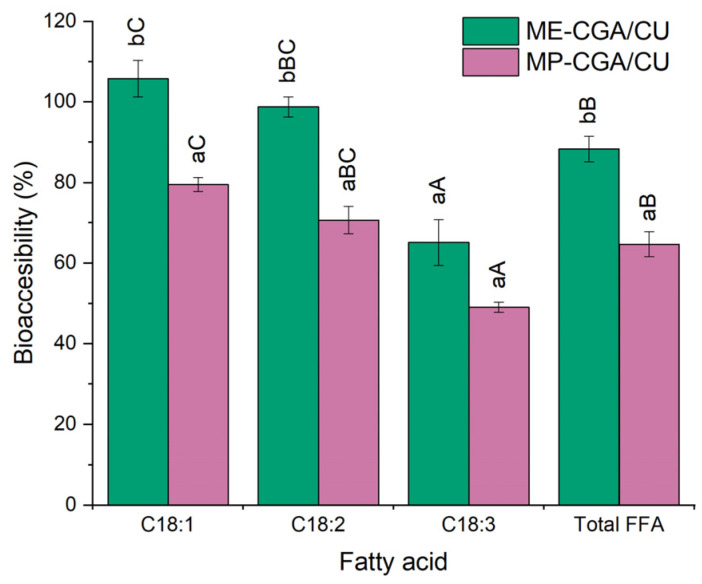
Bioaccessibility of total and major fatty acids (%) from LO in ME-CGA/CU and MP-CGA/CU. For each fatty acid, differences among samples (*p* ≤ 0.05) are marked with lowercase letters (a,b). Within a given sample, differences (*p* ≤ 0.05) in fatty acid bioaccessibility are denoted by uppercase letters (A–C).

MP-CGA/CU showed significantly lower CU bioaccessibility (62.7 ± 1.7%, *p* ≤ 0.05) compared with ME-CGA/CU (83.6 ± 6.1%), although this value can still be considered relatively high. This difference is consistent with the degree of lipolysis observed, since the generation of free fatty acids was significantly lower in MP-CGA/CU (64.7 ± 3.1%) than in ME-CGA/CU (88.3 ± 3.2%, *p* ≤ 0.05; Figure 6). This reduction can be explained by the larger oil droplets present in MP-CGA/CU after the gastric phase, which provided less surface area for pancreatic lipase activity and thereby decreased lipolysis. As a result, mixed micelle formation was limited, ultimately leading to reduced solubilization and bioaccessibility of CU. The high total fatty acid bioaccessibility observed in the MEs of this study (~88%) compared with previous work (~60%; [16]), where similar MEs with much larger droplet sizes (~25 µm vs. 2 µm) were evaluated, illustrates the relationship between droplet size and available surface area for lipase action, with smaller droplets facilitating enhanced lipid digestion. Figure 6 also shows that, in both ME-CGA/Cu and MP-CGA/CU, the bioaccessibility of the major fatty acids from LO decreased with increasing unsaturation. This trend, previously reported during the gastrointestinal digestion of LO-based MEs [15,16,57], can be attributed to fatty acid unsaturation, which affects both their hydrolysis efficiency by lipases and the resulting hydrophobicity that, in turn, determines their incorporation into micelles. Fatty acids with a lower degree of unsaturation are more readily hydrolyzed and, being more hydrophobic, are incorporated into micelles more efficiently [61].

## 4. Conclusions

This study demonstrated the feasibility of spray-dried LO-based MEs as co-delivery systems for CGA and CU. The process combining high-pressure homogenization and optimized spray drying conditions produced microparticles with consistently high oil encapsulation, ensuring structural stability and suitability for long-term use. Spray drying enhanced the retention of CGA by immobilizing it within the starch matrix, while CU encapsulation remained high despite a slight reduction linked to surface oil. Co-encapsulation did not negatively affect the EE of either compound, demonstrating the suitability of this system for the simultaneous protection of hydrophilic and lipophilic bioactives. The incorporation of antioxidants also contributed to improved oxidative stability, with both CGA and CU effectively delaying lipid oxidation, although without synergistic effects. Simulated gastrointestinal digestion highlighted the influence of compound nature on release behavior. CGA, completely released in the gastric phase, benefited from the protective effect of Capsul^®^, showing greater bioaccessibility in microparticles compared with liquid emulsions. In contrast, CU, mainly released in the intestinal phase, exhibited reduced bioaccessibility after spray drying, consistent with limited lipid digestion associated with larger oil droplets formed during drying and rehydration. In summary, spray-dried MEs proved effective for the co-delivery of hydrophilic and lipophilic bioactives, enhancing encapsulation and oxidative stability while providing relatively high bioaccessibility for both types of compounds. These findings support their potential for application in the design of functional foods enriched with diverse combinations of bioactive compounds.

## Figures and Tables

**Table 1:** D_4,3_ values (µm) and encapsulation efficiency (%) of CGA and CU in MEs.

	ME-C	ME-CGA	ME-CU	ME-CGA/CU
**D_4,3_ (µm)**	2.07 ± 0.02 ^a^	2.02 ± 0.04 ^a^	2.06 ± 0.03 ^a^	2.01 ± 0.04 ^a^
**EE of CGA (%)**	-	59.0 ± 0.9 ^a^	-	60.1 ± 0.3 ^a^
**EE of CU (%)**	-	-	99.9 ± 0.0 ^a^	99.8 ± 0.1 ^a^

EE: encapsulation efficiency; ME: multiple emulsion; CGA: chlorogenic acid; CU: curcumin; ME-C: ME without encapsulated bioactive compounds; ME-CGA: ME with CGA encapsulated in W_1_; ME-CU: ME with CU encapsulated in the oil phase; ME-CGA/CU: ME with CGA in W_1_ and CU in the oil phase. Values sharing the same letter (a) within a row are not significantly different (*p* > 0.05).

**Table 3:** Characterization of the mycroparticle systems.

Parameters	MP-C	MP-CGA	MP-CU	MP-CGA/CU
**EE of LO (%)**	90.1 ± 1.0 ^a^	94.6 ± 3.4 ^a^	96.8 ± 1.2 ^a^	95.2 ± 2.7 ^a^
**Total LO (mg/g)**	136.6 ± 0.1 ^a^	136.2 ± 0.2 ^a^	136.3 ± 0.4 ^a^	137.0 ± 0.2 ^a^
**IP (h)**	10.2 ± 1.4 ^a^	20.1 ± 0.1 ^b^	24.3 ± 0.5 ^c^	23.4 ± 0.9 ^bc^
**Moisture content (%)**	3.7 ± 0.2 ^a^	4.4 ± 0.0 ^b^	4.3 ± 0.2 ^b^	4.1 ± 0.0 ^ab^
**D_4,3_ (µm)**	8.4 ± 0.4 ^b^	10.5 ± 0.2 ^c^	13.0 ± 0.2 ^d^	7.2 ± 0.2 ^a^
**EE of CGA**	-	89.1 ± 0.6 ^a^	-	87.8 ± 1.5 ^a^
**EE of CU**	-	-	90.8 ± 1.2 ^a^	88.9 ± 0.6 ^a^

EE: encapsulation efficiency; LO: linseed oil; CGA: chlorogenic acid; CU: curcumin; IP: induction period; MP-C: microparticles without bioactive compounds; MP-CGA: microparticles with CGA in W_1_; MP-CU: microparticles with CU in the oil phase; MP-CGA/CU: microparticles with CGA in W_1_ and CU in the oil phase; Lowercase letters (a–d) within a row denote statistically significant differences (*p* ≤ 0.05) among samples.

## Data Availability

The original contributions presented in this study are included in the article and Supplementary Materials. Further inquiries can be directed to the corresponding author.

## Supplementary Materials

The following supporting information can be downloaded at https://www.mdpi.com/article/10.3390/antiox14101257/s1: Table S1: ANOVA for the EE of LO in MPs. Table S2: Composition of multiple emulsions (MEs). Figure S1: Scheme of multiple emulsion formulation. Figure S2: Particle size distribution of MEs and MPs determined by laser diffraction. Figure S3: SEM micrographs with particle size measurements of MPs: MP-C (**A**), MP-CGA (**B**), MP-CU (**C**), and MP-CGA/CU (**D**). Figure S4: Images of the powders obtained after spray-drying of MEs: MP-C (**A**), MP-CGA (**B**), MP-CU (**C**), and MP-CGA/CU (**D**).

## Abbreviations

The following abbreviations are used in this manuscript:

LO	Linseed Oil
ME	Multiple Emulsion
CGA	Chlorogenic Acid
CU	Curcumin
EE	Encapsulation Efficiency
OSA	Octenyl Succinic Anhydride
NaCas	Sodium Caseinate
PGPR	Polyglycerol Polyricinoleate
W_1_	Internal Aqueous Phase (first water phase)
W_2_	External Aqueous Phase (second water phase)
W/O/W	Water-in-Oil-in-Water Emulsion
O/W	Oil-in-Water Emulsion
ME-C	Multiple Emulsion without bioactives
ME-CGA	Multiple Emulsion with CGA in W_1_
ME-CU	Multiple Emulsion with CU in the oil phase
ME-CGA/CU	Multiple Emulsion with CGA in W_1_ and CU in the oil phase
D_4,3_	Volume-weighted Mean Diameter
UPLC	Ultra Performance Liquid Chromatography
UV/VIS	Ultraviolet/Visible Spectroscopy
CCD	Central Composite Design
RSM	Response Surface Methodology
ANOVA	Analysis of Variance
MP	Microparticle
MP-C	Microparticles without bioactives
MP-CGA	Microparticles with CGA in W_1_
MP-CU	Microparticles with CU in the oil phase
MP-CGA/CU	Microparticles with CGA in W_1_ and CU in the oil phase
IP	Induction Period
SEM	Scanning Electron Microscopy
SSF	Simulated Salivary Fluid
SGF	Simulated Gastric Fluid
