# Supplementary material for: Spray-Dried Multiple Emulsions as Co-Delivery Systems for Chlorogenic Acid and Curcumin
Source: Antioxidants (Basel). 2025 Oct 20;14(10):1257. doi: 10.3390/antiox14101257 (PMC12561490; doi:10.3390/antiox14101257)
Supplement: Supplementary file 1 [file antioxidants-14-01257-s001.zip › antioxidants-3878801-supplementary-figures.pdf]

Figure S1: Scheme of multiple emulsion formulation

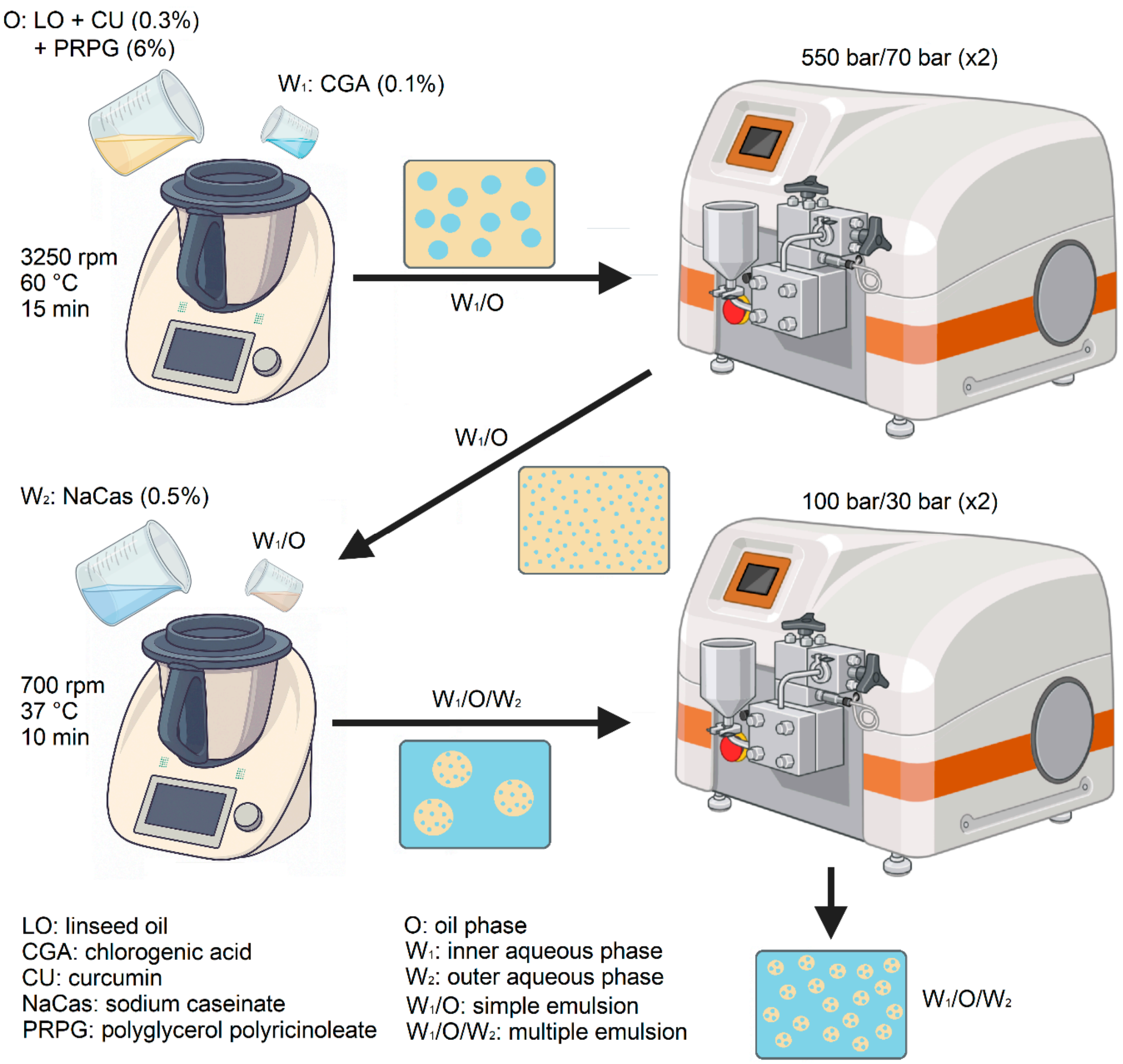

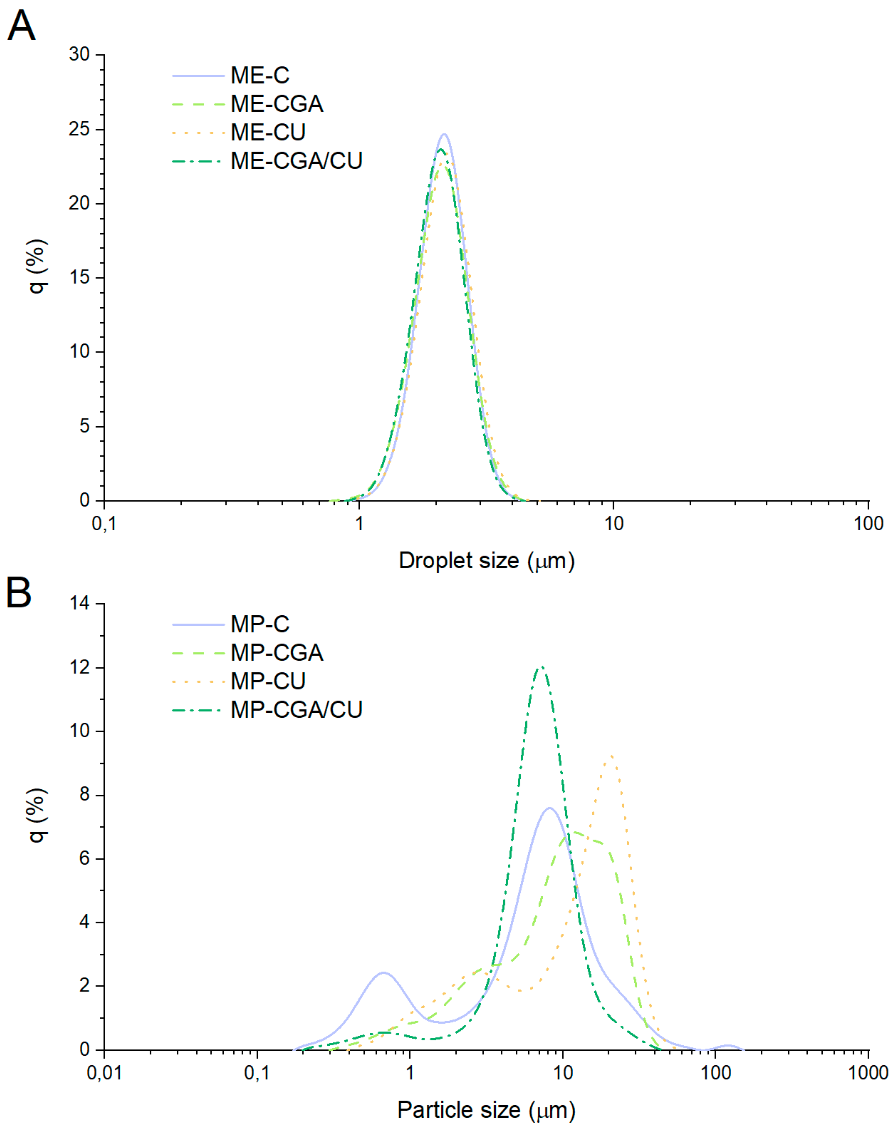

Figure S2: Particle size distribution of MEs (A) and MPs (B) determined by laser diffraction.

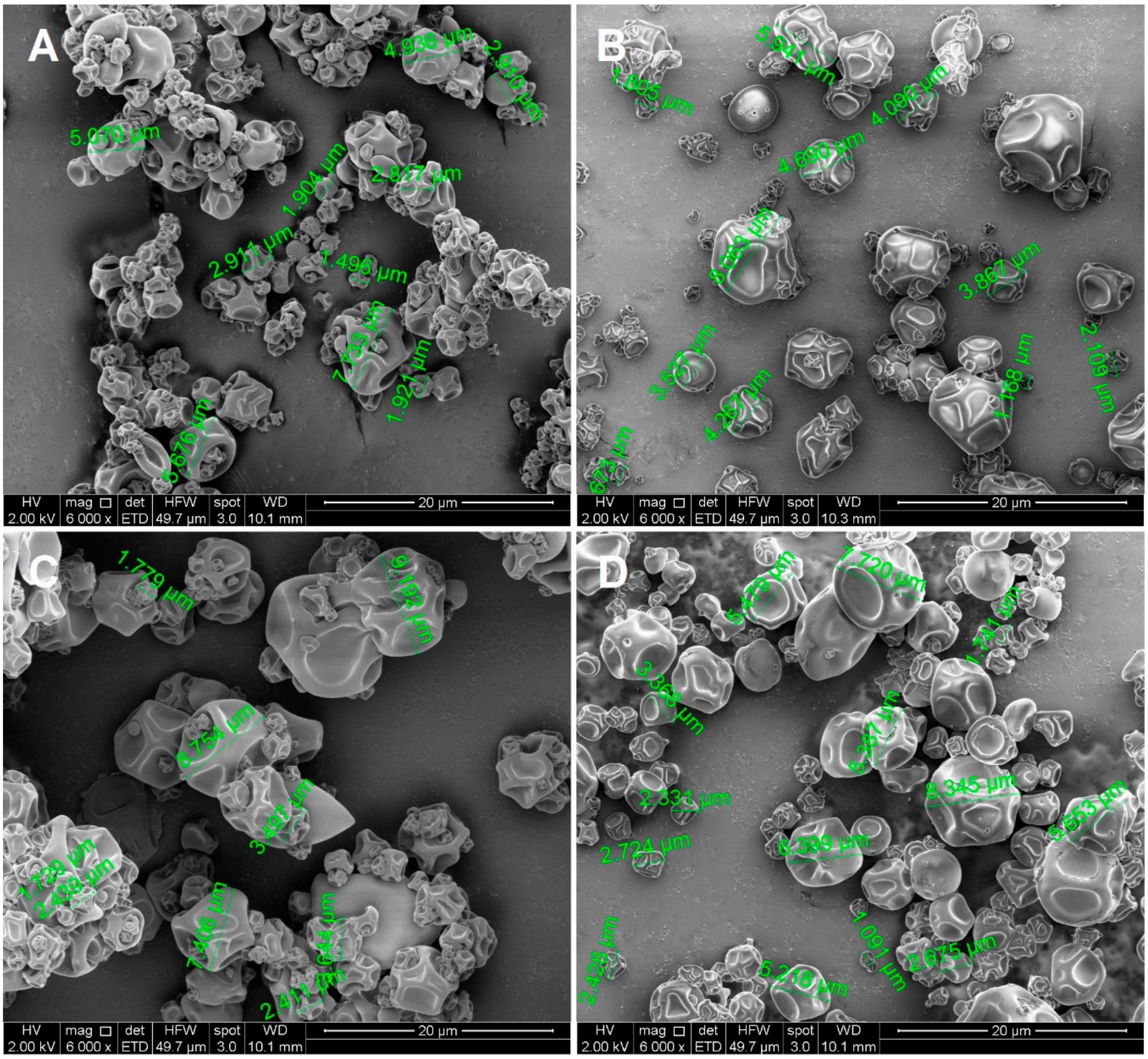

Figure S3: SEM micrographs with particle size measurements of MPs: MP-C (A), MP-CGA (B), MP-CU (C), and MP-CGA/CU (D).

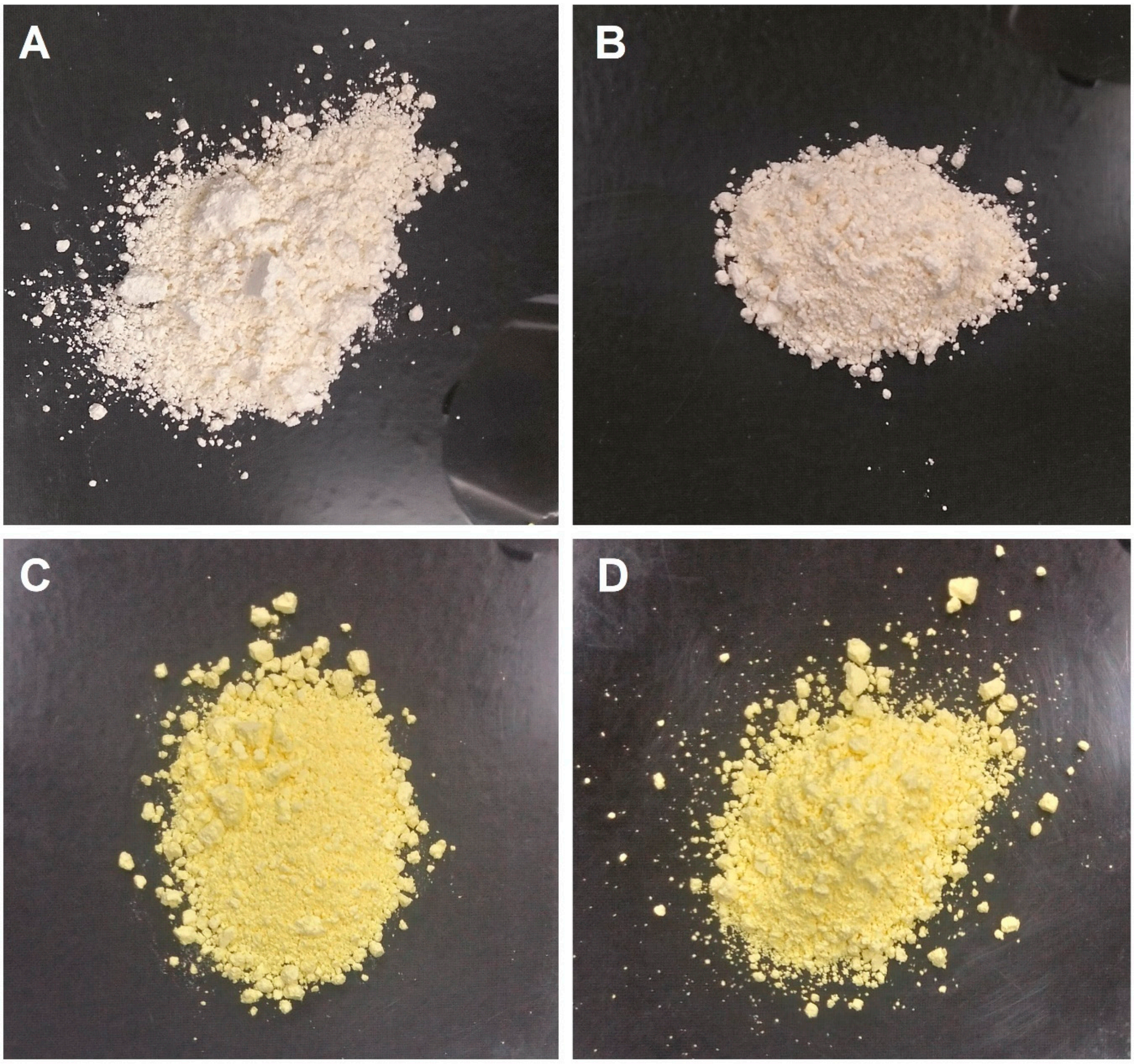

Figure S4: Pictures of powders obtained after spray drying process of MEs. MP-C (A), MP-CGA (B), MP-CU (C), and MP-CGA/CU (D).
