# Supplementary material for: Spray-Dried Multiple Emulsions as Co-Delivery Systems for Chlorogenic Acid and Curcumin
Source: Antioxidants (Basel). 2025 Oct 20;14(10):1257. doi: 10.3390/antiox14101257 (PMC12561490; doi:10.3390/antiox14101257)
Supplement: Supplementary file 1 [file antioxidants-14-01257-s001.zip › antioxidants-3878801-supplementary-tabls.pdf]

Table S1. Experimental design for microparticle formulation

Run	LO:Capsul® ratio	Inlet air temperature (°C)	EE of LO (%)
1	1:3.02	150	87.2 ± 0.4
2	1:6.28	150	93.6 ± 0.4
3	1:6	180	94.0 ± 0.8
4	1:3.3	180	91.8 ± 0.8
5	1:4.65	113.7	93.9 ± 0.8
6	1:3.3	120	86.6 ± 0.1
7	1:6	120	94.9 ± 0.9
8	1:4.65	186.3	95.0 ± 0.2
9	1:4.65	150	89.8 ± 0.3
10	1:4.65	150	89.3 ± 0.1
11	1:4.65	150	89.7 ± 0.7
12	1:4.65	150	91.2 ± 0.3

EE: Encapsulation efficiency; LO: Linseed oil

Table S2. ANOVA for the EE of LO in MPs

ANOVA	EE of LO	
	Regression coefficients	*p*-value
x_0	111.92	
x_1	7.598	0.0036*
x_2	-0.615	0.0817
$x_1 x_2$	-0.038	0.0347*
x_2^2	0.0027	0.0087*
Lack-of-fit		0.3926
R^2_{adj}		89.8%

EE: Encapsulation efficiency; LO: Linseed oil; x_1: LO:Capsul® ratio; x_2: inlet air temperature; x_1x_2: interaction between the LO:Capsul® ratio the inlet air temperature; R^2_{adj}: adjusted for degrees of freedom; *significant values ($p \leq 0.05$).
